# Molecular Identification of Atlantic Bluefin Tuna (*Thunnus thynnus*, Scombridae) Larvae and Development of a DNA Character-Based Identification Key for Mediterranean Scombrids

**DOI:** 10.1371/journal.pone.0130407

**Published:** 2015-07-06

**Authors:** Gregory Neils Puncher, Haritz Arrizabalaga, Francisco Alemany, Alessia Cariani, Isik K. Oray, F. Saadet Karakulak, Gualtiero Basilone, Angela Cuttitta, Salvatore Mazzola, Fausto Tinti

**Affiliations:** 1 Dept. of Biological, Geological and Environmental Sciences / Laboratory of Genetics and Genomics of Marine Resources and Environment (GenoDREAM), University of Bologna, Ravenna, Italy; 2 AZTI Tecnalia, Marine Research Division, Herrera Kaia, Pasaia, Gipuzkoa, Spain; 3 Instituto Español de Oceanografía, Centro Oceanográfico de Baleares, Palma, Spain; 4 Istanbul University, Faculty of Fisheries, Laleli, Istanbul, Turkey; 5 Laboratory of Molecular Ecology and Biotechnology, National Research Council, Institute for Marine and Coastal Environment (IAMC-CNR), Detached Unit of Capo Granitola, Trapani, Italy; Institut Maurice-Lamontagne, CANADA

## Abstract

The Atlantic bluefin tuna, *Thunnus thynnus*, is a commercially important species that has been severely over-exploited in the recent past. Although the eastern Atlantic and Mediterranean stock is now showing signs of recovery, its current status remains very uncertain and as a consequence their recovery is dependent upon severe management informed by rigorous scientific research. Monitoring of early life history stages can inform decision makers about the health of the species based upon recruitment and survival rates. Misidentification of fish larvae and eggs can lead to inaccurate estimates of stock biomass and productivity which can trigger demands for increased quotas and unsound management conclusions. Herein we used a molecular approach employing mitochondrial and nuclear genes (*CO1* and *ITS1*, respectively) to identify larvae (n = 188) collected from three spawning areas in the Mediterranean Sea by different institutions working with a regional fisheries management organization. Several techniques were used to analyze the genetic sequences (sequence alignments using search algorithms, neighbour joining trees, and a genetic character-based identification key) and an extensive comparison of the results is presented. During this process various inaccuracies in related publications and online databases were uncovered. Our results reveal important differences in the accuracy of the taxonomic identifications carried out by different ichthyoplanktologists following morphology-based methods. While less than half of larvae provided were bluefin tuna, other dominant taxa were bullet tuna (*Auxis rochei*), albacore (*Thunnus alalunga*) and little tunny (*Euthynnus alletteratus*). We advocate an expansion of expertise for a new generation of morphology-based taxonomists, increased dialogue between morphology-based and molecular taxonomists and increased scrutiny of public sequence databases.

## Introduction

Atlantic bluefin tuna (BFT, *Thunnus thynnus*) are the largest of tunas roaming the world's oceans and magnificent creatures that have fed and fascinated humankind for millennia. Vast herds of BFT ply the cold waters of the North Sea and North Atlantic Ocean for prey in the winter, returning to spawning grounds in the Mediterranean Sea and Gulf of Mexico in the spring and early summer. Fisheries have long taken benefit of these migratory patterns, slaughtering multitudes of tuna by hook, gaff, harpoon and net as they migrated through coastal waters to feed Roman Legions, burgeoning principalities, fishery empires and modern multinational corporations. This unrelenting appetite has recently brought stocks to the brink of collapse. Gone are the large BFT that spawned in the Black Sea and swelled the market places of Istanbul up until the mid 1980s [1,2]. BFT stocks that inspired a revolution in fishing gear in the North Sea, sustaining a French, Norwegian, German, Dutch and Danish fleet for several decades crashed in 1963 without warning [1]. In the 1960s, a massive shoal of BFT that appeared off the coast of Brazil was quickly targeted by Japanese long-liners and faded into the annals of history in as little as 7 years [3,4]. After many years of decline, the fisheries organization responsible for managing BFT stocks, the International Commission for the Conservation of Atlantic Tunas (ICCAT), finally enforced a rigorous recovery plan and stocks have started showing signs of recovery; although, the rate of recovery remains highly uncertain [5,6].

Currently, ICCAT manages BFT stocks as two populations: one which spawns in the Gulf of Mexico and forages in the North Western Atlantic (NWA) and a second that spawns in the Mediterranean Sea and forages in the Mediterranean and North Eastern Atlantic (NEA) [7]. Despite evidence suggesting that these two populations mix, ICCAT manages them separately, dividing their ranges along the 45° longitude. Novel insights developed from an array of technologies including satellites tags, genetics and microchemistry suggests that the population structure of BFT is much more complex [8–14]. If regional population structuring exists, it is paramount for the welfare of the species that it be maintained, in order to conserve genetic biodiversity and evolutionary potential. An accurate and confident model of the population structure of BFT and the factors that affect their distribution is key to their continued viability.

In the past 15 years, several molecular techniques have been used in an effort to develop a more accurate vision of BFT population structure and dynamics in line with the results developed by electronic tagging campaigns and traditional ecological knowledge (summary and references in [15]. Unfortunately, the results of these studies have been inconclusive and often contradictory. Due to the highly migratory nature of BFT, some research groups investigating the species' genetic population structure are now using only young tuna for their research as it is widely assumed that eggs, larvae and tuna of less than a few months age do not disperse far from their point of origin.

The location and abundance of early life stage fish is also used to improve stock assessments and our understanding of BFT spatial dynamics. Indices developed from the abundance of eggs and larvae collected from spawning sites have been used over 120 times to adjust and substantiate stock assessments of 18 different species within five teleost families throughout the world [16]. For decades, scientists from ICCAT member nations have been using larval indices generated from surveys conducted in the Gulf of Mexico to calibrate Virtual Population Analyses (VPAs) of western Atlantic BFT [17–19]. In 2013, the first standardized BFT larval indices for a Mediterranean spawning site were published based on larval surveys conducted by the Oceanographic Institute of Spain (IEO) around the Balearic Islands in the western Mediterranean [20]. Temporal shifts in BFT larvae abundance and condition can also provide important information about recruitment success, relative to short and long term environmental changes [21–23]. Surveys that have monitored the distribution of tuna larvae have shown that changes in relative abundances of different species are directly influenced by hydrodynamics [21,24–29]. In the context of a rapidly changing environment, our ability to properly identify and monitor fish species throughout their life history is critical for effective wildlife management and conservation efforts [30,31].

Unfortunately, early life stage fishes are often inaccurately identified by inexperienced technicians [32, 33]. These errors can lead to a misunderstanding of the spatial distribution of species, confusion over life history traits and population dynamics, inaccurate estimations of recruitment rates, survivorship and stock biomass, and potentially disguise the collapse or recovery of localized spawning sites [34,35]. The potential causes for misidentifications of tuna larvae are several: 1) Identification of tuna eggs and the larvae of some tuna species, using morphological characteristics alone, is very challenging, requiring an in depth knowledge of taxonomy, patience and experience [36–38], 2) samples are often badly damaged during collection or as a result of preservation [39–41], 3) some tuna identification keys are inaccurate and require updating, and 4) expert taxonomists in general are few and in demographic decline [42–44].

Due to the difficulties of identifying tuna larvae researchers occasionally outsource the task to distant laboratories such as the Sea Fisheries Institute, Plankton Sorting and Identification Center in Poland [17,19,22,45]; however, misidentification of larvae has occurred at that facility in the past as well (F. Alemany personal communication). Others have simply resorted to assigning scombrids to lower taxonomic levels [22,46]. Advances in molecular techniques and genetic barcoding now offer another solution to this problem.

There are a variety of ways in which researchers using genetic barcodes analyze their data in order to identify their specimens. The most commonly used tools for sequence association in barcoding studies are: 1) alignment with voucher sequences from online databases using Basic Local Alignment Search Tool (BLAST) tools provided by National Center for Biotechnology Information or the ID system provided by the Barcode of Life Database (BOLD), 2) Neighbour-Joining trees, and 3) classification using a molecular key of characteristic attributes. The BLAST program uses a heuristic algorithm to identify the sequences contained in GenBank that are most similar to the query sequence provided by the user [47]. The ID System by BOLD employs a Hidden Markov Model (HMM) for alignment construction and returns only sequence matches that are less than 1% divergent from the query sequence [48]. Neighbour-Joining (NJ) trees are ubiquitous among barcoding publications. They use a hierarchical clustering method to construct a phenogram based on a distance matrix of similarity between reference and query sequences. These distance matrices can be constructed by various methods which can impact species identification accuracy and measures of confidence. The Kimura 2-parameter model (K2P) for NJ trees has become the default model for most fish species identification studies; however, researchers have recently been challenging this assumption [49–53].

This study describes how larvae collected from the Strait of Sicily, Western Ionian Sea and Levantine Sea were acquired from three different institutions for genomic analysis within ICCAT's Atlantic wide research programme for bluefin tuna (GBYP). All larvae had been provisionally identified as *Thunnus thynnus* by technicians using morphology-based methods. All larvae were barcoded using a 650bp fragment of the *CO1* gene and identified to species in an effort to assess the accuracy of identification. We have also compared the effectiveness of various methods used for associating sample sequences to reference or voucher sequences. We review the overall effectiveness of the Neighbour-Joining tree approach and compare two methods used for distance matrix construction (p-distance vs. K2P). Finally, we develop and assess a character-based key which uses unique genetic characteristics in much the same way as taxonomic keys that identify organisms based on diagnostic morphological features.

## Materials and Methods

### Sample collection

#### Strait of Sicily

**Fig 1:**
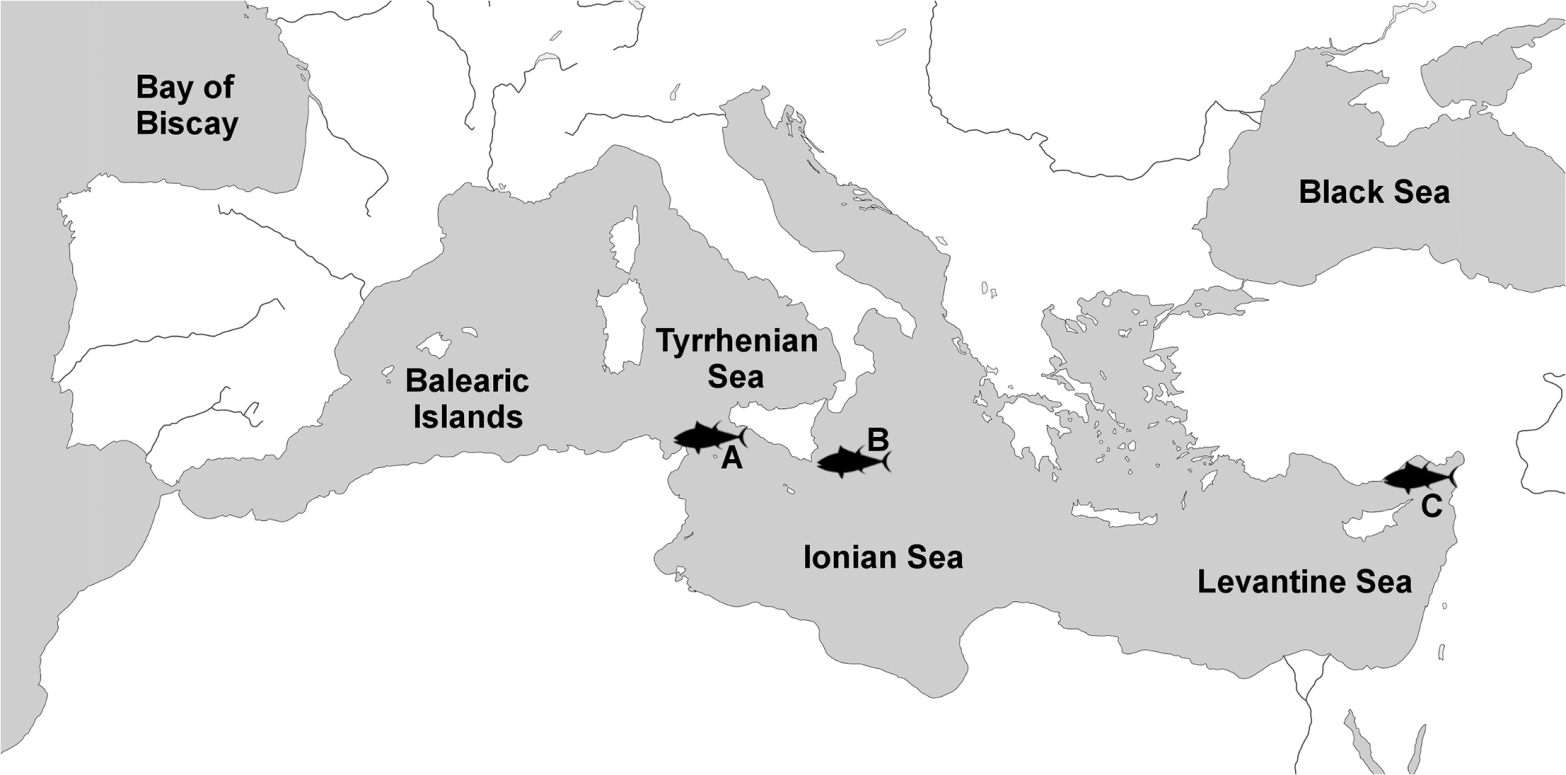
Map of the Mediterranean Sea and surrounding area with three larvae sampling sites: A) Strait of Sicily, B) Ionian Sea and C) Levantine Sea.

Larval tows were performed by Istituto per l’Ambiente Marino Costero of the National Research Council of Italy (IAMC-CNR) in the waters off Sicily’s southern coast (35°30’N-38°90’N, 12°38’E-15°10’E; Fig 1[A]), on board the R/V “Urania”, during 17–21 July 2011 and 5–19 July 2012 using two Bongo nets with 40 and 90cm diameters equipped with 1 mm black mesh. Bongo nets were towed obliquely from the surface to 100 m and back to the surface at two knots. All larvae were preserved in 96% ethanol and transported to the laboratory where they were identified to family, genus or species level when possible; using various taxonomic keys [54–58]. A total of 88 larvae with a mean length of 4.9 ± 1.5 mm and provisionally identified as tunas were sent to the GenoDREAM laboratory at the University of Bologna for genetic barcoding. An additional 4 non-scombrid larvae were also provided as outliers.

#### Capo Passero, Ionian Sea

OCEANA’s 2008 larval survey aboard the R/V Marviva Med was the first BFT larval survey undertaken by an NGO team in the Mediterranean [26]. Larval tows were conducted 15 July—11 August 2008 using a bongo 90 net with a quadrangular mouth opening equipped with 500 μm mesh for horizontal surface plankton tows east of Sicily (36°30’N-37°33’N, 15°35’E-15°59’E; Fig 1[B]). The net was towed at 2–2.5 knots at the surface for a constant duration of 10 minutes. Upon retrieval of nets from the sea, all larvae were immediately preserved in ethanol. All larvae were then dispatched to experts at IEO facilities in Palma de Mallorca where they were identified to species by experts. A total of 58 larvae identified as *T*. *thynnus* were dispatched to the GenoDREAM laboratory in November 2013 for genetic analysis.

#### Levantine Sea

A larval cruise was conducted by Istanbul University during 20–24 June 2012 along the southern coast of Turkey (36°07’N-36°10’N, 33°33’E-33°47’E; Fig 1[C]). Larvae were collected from surface waters using a Bongo net of 90 cm diameter and 1 mm mesh size, towed at 2–2.5 knots for 10 minutes. All captured larvae were immediately preserved in 96% ethanol and identified to species level on-board using microscopes and the taxonomic key by Richards (2005). A total of 38 larvae with a mean length of 6.9 ± 1.5 mm were conditionally identified as *T*. *thynnus*. These larvae were then bisected and the caudal sections were dispatched to GenoDREAM lab for genetic verification.

### Ethics statement

All larvae used in this study were collected from the Mediterranean Sea using plankton nets and sacrificed via immersion in 96% ethanol, according to standard larval survey practices. Permission to conduct the larval surveys and all legal permits were granted by the International Commission for the Conservation of Atlantic Tuna (ICCAT) and the General Fisheries Commission for the Mediterranean. ICCAT is the intergovernmental organization responsible for the management of bluefin tuna in the Atlantic Ocean and Mediterranean Sea and all research was coordinated through their “Atlantic-wide research programme for bluefin tuna” (GBYP). ICCAT issued a recommendation (Rec. 11–06) allowing the parties involved in this research to collect and sacrifice larvae for the purposes of genetic research as well as ship samples from one country to another (Certificate No. ICCAT RMA12-049).

### DNA extraction

All 188 scombrid and non-scombrid larvae were digested overnight in a Proteinase K solution and genomic DNA was extracted from each and purified using Promega’s Wizard SV96 Genomic DNA Purification kit and vacuum manifold. Fragments of the *CO1* gene (~ 650 bp) were amplified (PCR) using FishF2 (5’-TCGACTAATCATAAAGATATCGGC- AC-3’) and FishR2 (5’-ACTTCAGGGTGACCGAAGAATCAGAA-3’) primers first published by Ward et al. [59]. PCR reactions were performed in 50 μL volume consisting of 1x PCR Buffer, 1.0 μM of each primer, 160 μg/mL of BSA, 0.4 mM of dNTPs, 1.5 mM of MgCl_2_, 2.5 U/mL of Invitrogen Taq polymerase and ~100 ng of template DNA. PCR conditions consisted of 94°C for 3 min, 35 cycles of 30 sec at 94°C, 30 sec at 52°C, and 30 sec at 72°C, with a final extension at 72°C for 3 min. Due to the introgression of the mitochondrial genome of *T*. *alalunga* into the *T*. *thynnus* gene pool [41,60–63], all larvae that were identified as *T*. *alalunga* using the *CO1* gene were then barcoded using sequences from the *ITS1* region. DNA extractions from archived tissue samples of adult *T*. *thynnus* (2) and *T*. *alalunga* (2) were used as reference standards for all further analyses. Fragments of the *ITS1* gene (~680bp) were amplified using the *ITS1*F (5’-TCCGTAGGTGAACCTGCGG-3’) and *ITS1*R (5’-CGCTGCGTTCTTCATCG-3’) primers designed by Chow et al. [61]. All other PCR reagents were the same as above and conditions were as follows: 94°C for 3 min, 35 cycles of 30 sec at 94°C, 30 sec at 50°C, and 30 sec at 72°C, with a final extension at 72°C for 3 min.

After receiving all *CO1* sequences from Macrogen Europe (Amsterdam, Netherlands), they were aligned in MEGA6 using the ClustalW algorithm and trimmed to 612bp.

### BLAST and BOLD search engines

All larval sequences were converted to FASTA format and submitted to a nucleotide BLAST through the NCBI website (http://www.ncbi.nlm.nih.gov/). The first five sequence matches with highest similarity to database references (max ident) were analyzed for species consistency. Similarly, all sequences were submitted to BOLD's Identification System (http://www.boldsystems.org/) for comparison with all species level barcode records of animals (148,815 sequences as of 09 Nov 2014). All matches were analyzed using the percent similarity score.

### Neighbour joining tree analysis

Ten reference sequences from each of thirteen scombrid species were downloaded from BOLD (n = 122) and GenBank (n = 8) (S1 Appendix). Sequences were mined from GenBank only when the number of sequences on BOLD were insufficient. Aside from the BFT reference sequences, eight of the species occur in the Mediterranean Sea and may have larvae associated with those of BFT (*Auxis rochei*, *Auxis thazard*, *Scomber colias*, *Scomber scombrus*, *Euthynnus alletteratus*, *Katsuwonus pelamis*, *Sarda sarda*, *T*. *alalunga*,*)*. The remaining four species are closely related to the other species and were included in order to rule out the occurrence of genetic introgression (*Scomber japonicus*, *Thunnus albacares*, *Thunnus maccoyii*, *Thunnus atlanticus*). Reference sequences were sourced from throughout each species’ geographic distribution in order to have ample representation of genetic variation. Various phenograms (all sequences, only reference sequences, all larvae and reference sequences belonging to Mediterranean species) were built using the Neighbour Joining method [64] with both the Kimura-2-parameter (K2P) distance model [65] and p-distances [66] for distance matrix construction. The statistical support of each node was tested using bootstrap analysis [67] with 1000 replications in MEGA6 [68]. Selected trees were modified using FigTree (http://tree.bio.ed.ac.uk/software/figtree/).

### Assignment by characteristic attribute key

All variable sites of an alignment containing all reference and larvae sequences were highlighted using MEGA6. This simplified alignment was inspected for loci containing unique nucleotides that were diagnostic of particular species. The character-based key constructed here differs from those constructed by [69] in that it requires first assignment to genus and then species. In this way we have used nucleotides that may not be unique to single species or genera. Rather we are providing a multi-step assignment tool which uses multiple characteristics, much like a taxonomic key. According to the terminology used by [68], we have selected “pure characteristic attributes” (PCAs), or specific nucleotides located at variable sites that are unique to single clades and thus diagnostic for single clades. Some of these characteristic attributes stand alone and are called “simple CAs”, whereas other loci should be used in combination and are thus called “compound CAs”. Once the key was constructed all larval sequences were assigned to species accordingly.

## Results

DNA extractions from all larvae were successfully amplified, sequenced and analyzed. Sequences from 84 larvae with photographic records were uploaded to the Barcode of Life Database (Project: MLRV; Accession numbers: MLRV001-15 to MLRV084-15). All other sequences were uploaded directly to the GenBank database (KT003822 to KT003924). A few unexpected challenges were encountered concerning the reference sequences downloaded from both BOLD and GenBank. One *T*. *thynnus* reference sequence (BOLD: GBGCA443-10; GenBank: GQ414572) affiliated with the work of [62] corresponds with *CO1* sequences of *T*. *alalunga*. Within that work, the authors clearly state that this sequence belongs to a BFT with introgressed mtDNA from albacore; however, no mention of this is associated with the sequences itself in either database. They also describe how two sequences belonging to Pacific-like *T*. *thynnus* (Atlantic bluefin with introgressed *T*. *orientalis* mitochondria) were used and published in GenBank (GQ414570 and GQ414573). Both of these sequences were later incorporated into the BOLD database (GBGCA445-10 and GBGCA442-10, respectively); however, in both databases GQ414570 is identified as *T*. *thynnus*, while QQ414573 is identified as *T*. *orientalis*. Another sequence featured in [62], which they referred to as *T*. *orientalis*, has since been uploaded to GenBank (GQ414566) and BOLD (GBGCA449-10) under the title of *T*. *thynnus*. BOLD and BLAST queries, as well as our CA key, clearly identify this as a *T*. *orientalis* sequence. It is likely that the authors have confused sequences and their IDs at some point (confirmed by J. Viñas). Finally, a single *T*. *orientalis* sequence (GenBank: JN097817; BOLD: GBGCA1390-13) uploaded by several researchers from the South Korean National Fisheries Research and Development Institute matches those of *T*. *thynnus*.

### BLAST assignment

For all but two sequences, BLAST provided a species match with an identity similarity higher than 99% (Table 1). The median value for maximum similarity scores across the type five selected matches for each larva was 100%. The remaining two larvae from the Strait of Sicily received identity similarity scores of 93% and 95% for sequences belonging to bullet tuna (*Auxis rochei*, Scombridae) and *T*. *thynnus*, respectively. Overall, only 42% of larvae were identified as *T*. *thynnus*. In fact, nearly as many (39%) were identified as bullet tuna. The larvae collected in the Strait of Sicily were composed of five different scombrid species with only 21 identified as *T*. *thynnus*. Although the samples from the Levantine Sea contained fewer taxa, none of the larvae provided were identified as *T*. *thynnus*. All larvae collected from the Ionian Sea, offshore from Capo Passero were identified as *T*. *thynnus* and received identity similarity scores of 100%. The four non-scombrid larvae from Sicily were identified as *Chromis sp*., picarel (*Spicara smaris*, Centracanthidae), greater weever (*Trachinus draco*, Trachinidae), and pygmy lanternfish (*Lampanyctus pusillus*, Myctophidae). Five larvae from the Levantine Sea were assigned to two additional outlier taxa: brown comber (*Serranus hepatus*, Serranidae) (2) and common pandora (*Pagellus erythrinus*, Sparidae) (3). All larvae provisionally identified as albacore using *CO1* sequences and later barcoded with the *ITS1* gene clustered with *T*. *alalunga* standards (data not shown), thereby ruling out the possibility of false species identification due to hybridization/mtDNA introgression.

**Table 1:** Species and origin of larvae identified using *CO1* and *ITS1* genetic markers, BLAST neighbour-joining reconstruction and character-based assignment.

Species	Strait of Sicily	Capo Passero	Levantine Sea
*Auxis rochei*	53	0	21
*Eythynnus alleteratus*	2	0	12
*Scomber japonicus*	1	0	0
*Thunnus alalunga*	11	0	0
*Thunnus thynnus*	21	58	0
Non-scombrid larvae	4	0	5
Total	92	58	38

### BOLD assignment

All larvae were identified by BOLD with a confidence score of 99–100%, aside from the two individuals collected the Strait of Sicily discussed above for which a “no match” was returned. No match was given by BOLD because of the program's 1% divergence threshold. All other specimen identifications were equal to those provided by BLAST.

### Neighbour-joining tree analysis

**Fig 2:**
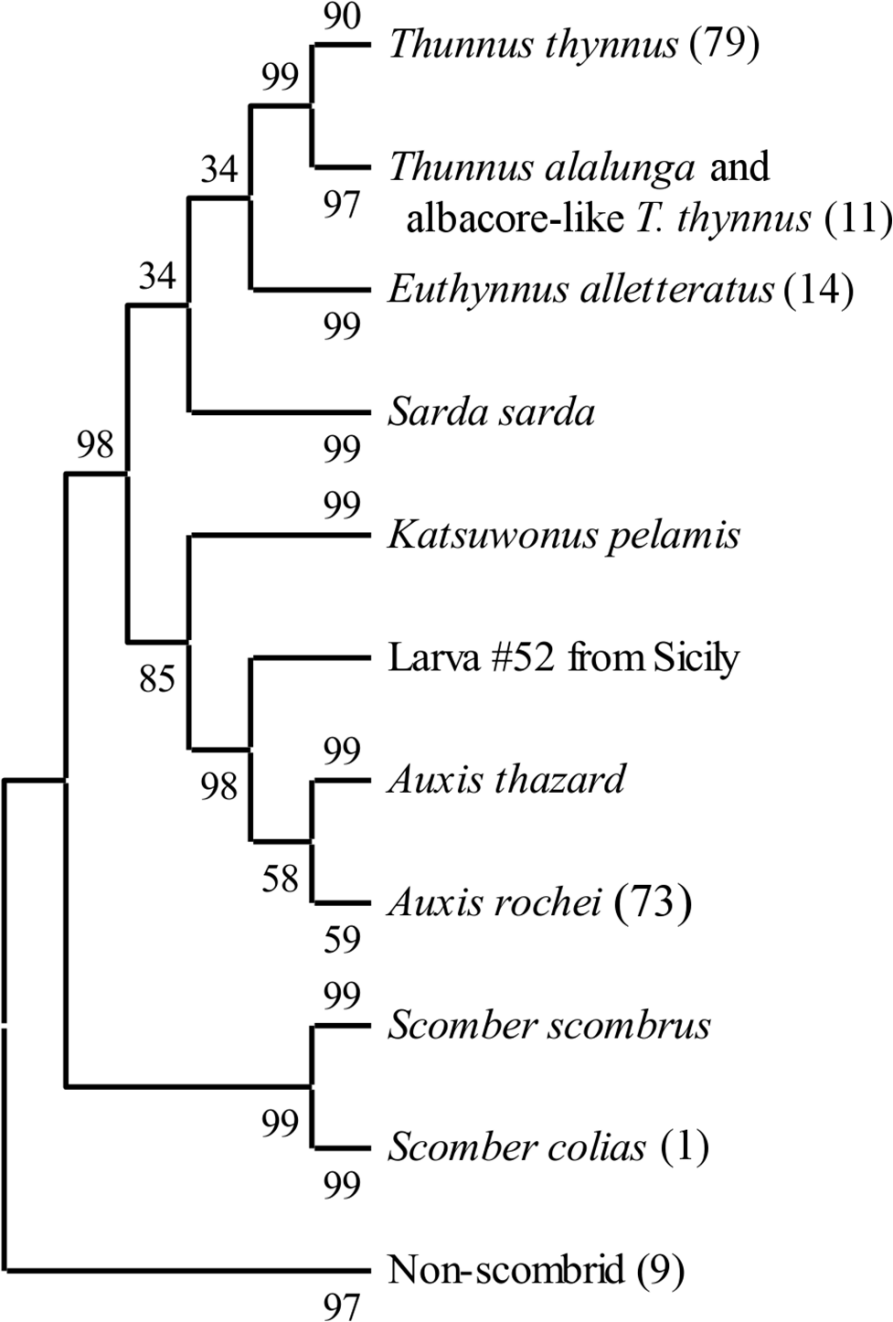
Neighbour-joining phenogram of Mediterranean scombrid reference sequences clustered with number of unknown larvae in parentheses. The percentage of replicate trees in which the associated taxa clustered together in the bootstrap test (1000 replicates) are shown next to the branches [67]. The tree is drawn to scale, with branch lengths in the same units as those of the evolutionary distances used to infer the tree. The evolutionary distances were computed using the p-distance method [66] and are in the units of the number of base differences per site. The analysis involved 280 nucleotide sequences. All ambiguous positions were removed for each sequence pair. There were a total of 612 nucleotide positions in the final dataset.

**Fig 3:**
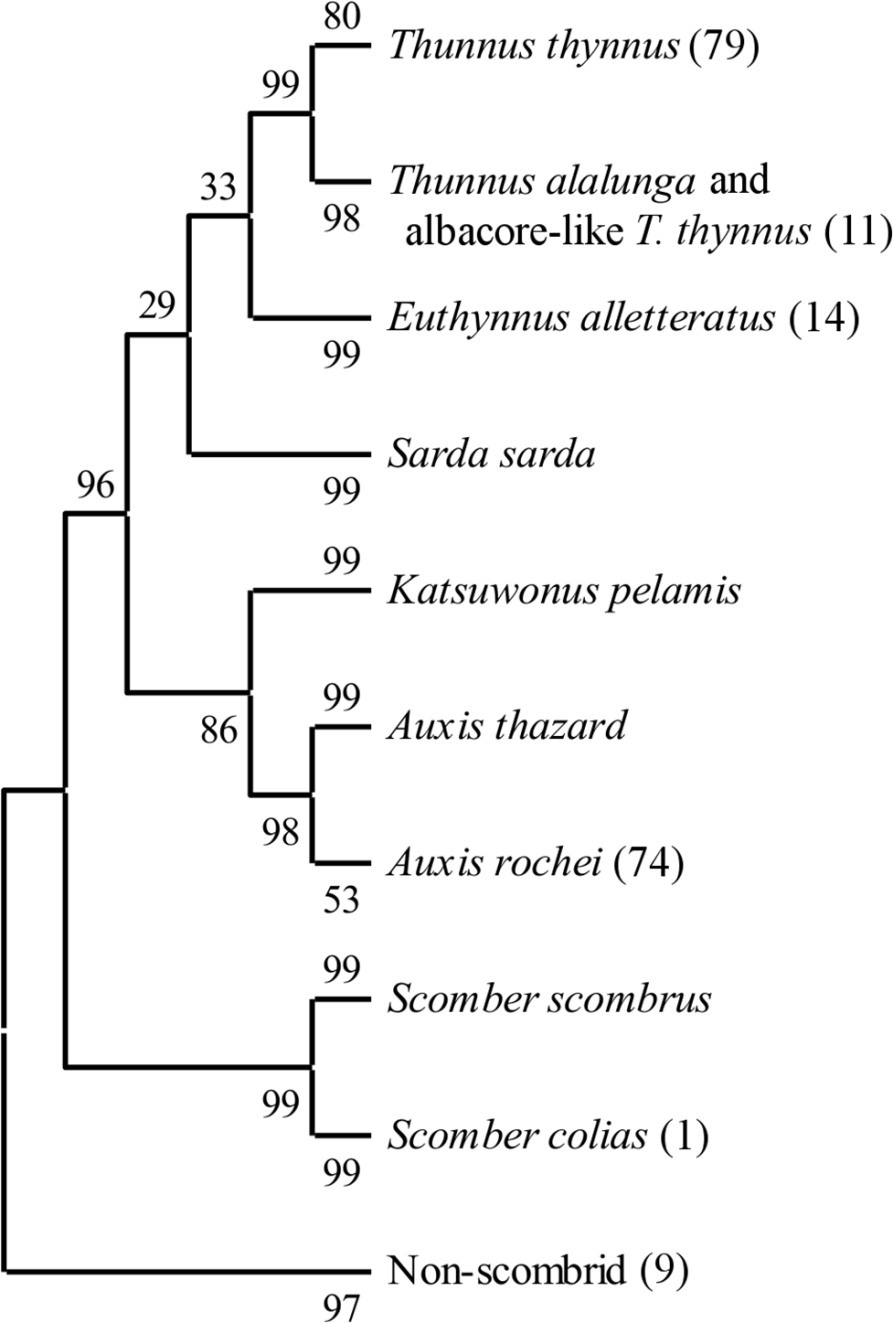
Neighbour-joining phenogram of Mediterranean scombrid reference sequences clustered with number of unknown larvae in parentheses. The percentage of replicate trees in which the associated taxa clustered together in the bootstrap test (1000 replicates) are shown next to the branches [67]. The tree is drawn to scale, with branch lengths in the same units as those of the evolutionary distances used to infer the tree. The evolutionary distances were computed using the Kimura 2-parameter model [65] and are in the units of the number of base differences per site. The analysis involved 280 nucleotide sequences. All ambiguous positions were removed for each sequence pair. There were a total of 612 nucleotide positions in the final dataset.

**Fig 4:**
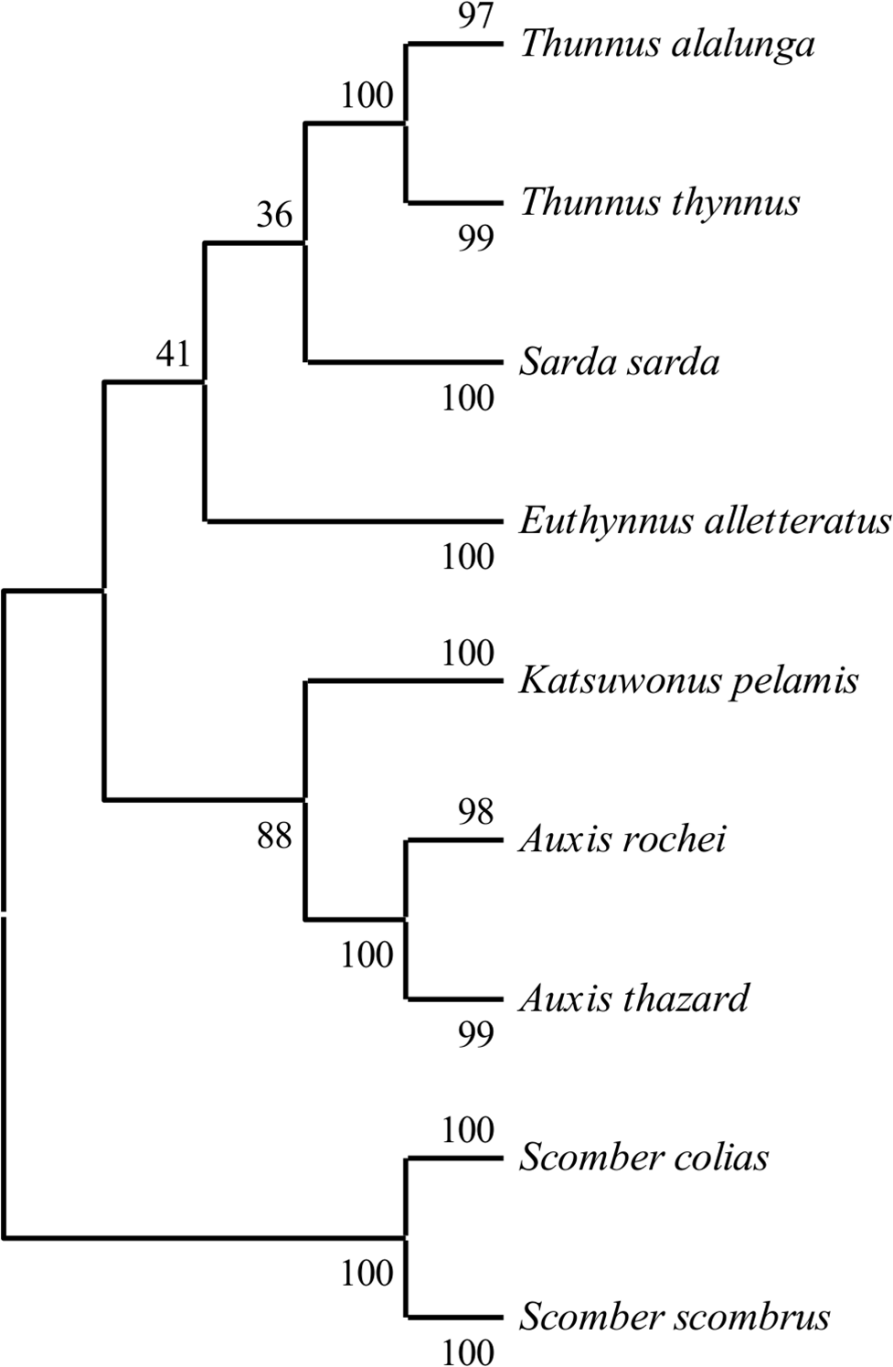
Neighbour-joining phenogram of Mediterranean scombrid reference sequences only. The percentage of replicate trees in which the associated taxa clustered together in the bootstrap test (1000 replicates) are shown next to the branches [67]. The tree is drawn to scale, with branch lengths in the same units as those of the evolutionary distances used to infer the tree. The evolutionary distances were computed using the p-distance method [66] and are in the units of the number of base differences per site. The analysis involved 91 nucleotide sequences. All ambiguous positions were removed for each sequence pair. There were a total of 612 nucleotide positions in the final dataset.

**Fig 5:**
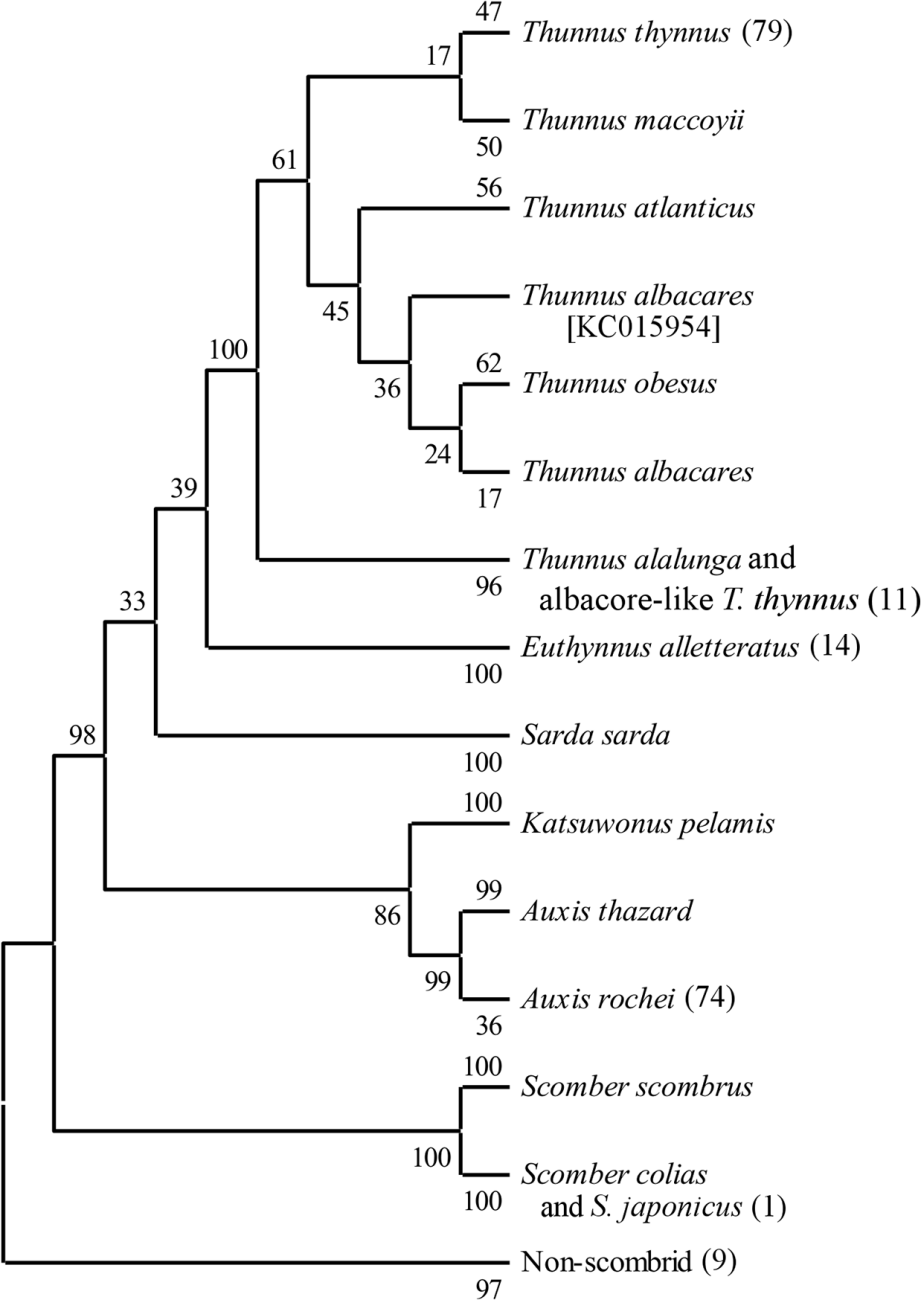
Neighbour-joining phenogram of reference sequences (including non-Mediterranean *Thunnus* species) clustered with number of unknown larvae in parentheses. The percentage of replicate trees in which the associated taxa clustered together in the bootstrap test (1000 replicates) are shown next to the branches [67]. The tree is drawn to scale, with branch lengths in the same units as those of the evolutionary distances used to infer the tree. The evolutionary distances were computed using the p-distance method [66] and are in the units of the number of base differences per site. The analysis involved 330 nucleotide sequences. All ambiguous positions were removed for each sequence pair. There were a total of 612 nucleotide positions in the final dataset.

Neighbour joining tree analysis using *CO1* sequences produced well-defined clusters of candidate larvae with reference sequences (Figs 2 and 3). All larvae clustered with reference taxa in the same manner as the BLAST and BOLD results. Phenograms containing only the larvae and reference sequences for species found in Mediterranean Sea showed lowest bootstrap probability (BP) for branch nodes separating the true tunas (BFT and albacore) from the other scombrids (BP = 33–35) and a clade containing the true tunas and *E*. *alletteratus* from the other species (BP = 30–34). The node containing the *A*. *rochei* reference sequences and 74 larvae was the least stable with BPs of 53–59. All other nodes had BPs > 80. Phenograms based on distance matrices using p-distances (Fig 4) had consistently higher BP values than those built with K2P distances (Fig 3). P-distance based phenograms also tended to exclude larval sequences from clusters, as is the case with one larva in the Auxis spp. cluster in Fig 2. When the larval sequences were removed from the alignments, all BP values increased (Fig 4). Predictably, BP values for the A. rochei (ΔBP = 39) and T. thynnus (ΔBP = 17) nodes increased most dramatically, as they were the two clusters to increase in sequence size most dramatically. When *T*. *atlanticus*, *T*. *maccoyii*, *T*. *obesus* and *T*. *albacares* reference sequences were included in the alignments, the neighbour-joining tree based on p-distances failed to differentiate clusters for each and combined all with the same cluster alongside the T. thynnus and related larvae sequences (BP = 61; Fig 5). The topology of this same clade in the corresponding K2P-based phenogram changed somewhat but the composition remained the same, albeit the BP value was much lower (BP = 35).

The *ITS1* NJ tree constructed from sequences of voucher specimens and larvae identified as *T*. *alalunga* using *CO1* did not reveal any introgressed BFT.

### Character-based assignment

Construction of a character-based key uncovered 68 nucleotides capable of distinguishing all reference taxa. As the primary purpose of our research was the isolation of *T*. *thynnus* larvae from the rest of the catch, *Thunnus spp*. sequences were the first to be isolated using four diagnostic nucleotides (Table 2). Using these nucleotides a total of 90 larvae were identified as members of the *Thunnus* genus, representing less than half of all larvae. Six bases separating *T*. *alalunga* reference sequences from the other five *Thunnus* species identified eleven albacore tuna among the larvae. A single thymine located at position 231 of our alignment was capable of isolating all *T*. *thynnus* references from the other species of the same genus. When these criteria were applied to the larvae, a total of seventy-eight larvae were identified as bluefin tuna. One outstanding individual was identified as *T*. *maccoyii*, *T*. *atlanticus*, *T*. *albacares or T*. *obesus* using these criteria. Since none of these species can be found in the Mediterranean Sea, this is likely an example of genetic introgression. Three bases, found to be discriminatory for the *Auxis* species of the Mediterranean, identified seventy-four larvae among the remaining samples. All of these *Auxis* candidate larvae were identified as *A*. *rochei* using eight bases that were found to discriminate between *A*. *rochei* and *A*. *thazard* reference sequences. Using six diagnostic loci, fourteen *Euthynnus alletteratus* larvae were identified. Eleven characteristic loci set the *Scomber spp*. apart from the other species and ten loci were used to identify a single larva. Six diagnostic bases were found for *Katsuwonus pelamis* and *Sarda sarda* but none of the larvae analyzed were identified as members of either species. Since the character-based identification key was developed for the identification of scombrids only, the remaining nine larvae could not be identified.

**Table 2:** Characteristic attributes for capable of distinguishing taxa of scombrids in the Mediterranean Sea.

Taxa	Diagnostic nucleotides
*Thunnus* spp.	327[A], 372[G], 525[T], 540[T]
*Thunnus alalunga*	228[T], 273[G], 438[C], 495[T], 606[G], 609[T]
*Thunnus thynnus*	231[T]
*T. thynnus, T. albacares, T. maccoyii, T. obsesus, T. atlanticus*	228[C], 273[A], 495[C], 606[A], 609[C]
*Auxis* spp.	393[T], 453[T], 456[C] (all 3 must be part of the package).
*Auxis rochei*	225[T], 247[T], 315[C], 336[T], 348[C], 465[A], 468[A], 486[T]
*Auxis thazard*	225[C], 247[C], 315[T], 336[C], 348[T], 465[G], 468[G], 486[C]
*Euthynnus alletterratus*	303[G], 312[A], 408[G], 426[G], 498[G], 553[T]
*Scomber spp.*	81[T], 127[G], 210[A], 235[C], 249[G], 258[G], 260[C], 351[C], 393[C], 434[G], 519[T]
*Scomber colias/japonicus*	93[C], 192[T], 225[G], 240[G], 306[C], 312[G], 321[A], 342[T], 414[C], 423[A], 436[G], 438[A], 561[T], 612[C]
*Scomber scombrus*	67[G], 72[T], 129[C], 240[A], 303[A], 306[A], 321[G], 507[G], 543[T], 546[T]
*Sarda sarda*	216[T], 258[T], 264[C], 279[G], 543[G], 567[T]
*Katsuwonus pelamis*	366[T], 378[T], 390[A], 501 [G], 555[G], 582[T]

Position of each variable nucleotide is given in relation to 612bp alignment of all sequences. Diagnostic nucleotides at each locus are given in parentheses.

## Discussion

### (Mis)Identification of larvae

The 11 species identified among the samples are typical of plankton surveys conducted in the region and during this sampling season. BFT larvae are commonly associated with larvae of other scombrids, having been previously captured with dense concentrations of bullet tuna off the coasts of Tunisia [70,71], Sicily [26] and the Balearic Islands [72]. They have also been found alongside albacore tuna [21,26] and Atlantic black skipjack (*Euthynnus alletteratus*, Scombridae) [21]. Among these species, BFT are found in proportionally higher concentrations in deep offshore waters beyond shelf breaks [25,70]. Researchers in Italy [71] captured *L*. *pusillus* larvae in the Strait of Sicily in June-July 2000. Larvae of *S*. *hepatus*, *Chromis chromis* (Pomacentridae), *P*. *erythrinus* and *T*. *draco* have been captured in the Aegean during June of 2003–2006 [73]. Alemany et al. [25] have encountered all the species that we have identified in the Balearic Islands, aside from *P*. *erythrinus* and Atlantic chub mackerel (*Scomber colias*, Scombridae). Accurate identification of these samples to species level is a testament to the versatility of the genetic marker used and the potential of the ever-expanding resources of the Barcode of Life Database.

The high number of correctly identified BFT larvae within the Sicilian samples was expected, since large quantities *of T*. *thynnus* are typical of the Sicilian Channel, western Ionian and southern Tyrrhenian Seas [74,75]. To date, BFT larvae have been found in highest concentration in Mediterranean waters around Sicily, the Balearic Islands and the southern coast of Turkey [23,24,76,77]. It comes as no surprise that all 58 larvae provided by the IEO and OCEANA-Marviva project were correctly identified, since their staff are leading experts in the field of ichthyoplanktology. However, the fact that no BFT larvae were provided amongst the 38 larvae received from the Levantine Sea calls into question all previous publications on the subject of BFT reproduction in that area. For example, Oray and Karakulak [78] captured 121 bluefin tuna (*T*. *thynnus*), 94 bullet tuna (*A*. *rochei*) and 22 Atlantic black skipjack (*E*. *alletteratus*) larvae in the northern Levantine basin during larval surveys conducted during 5–18 June 2004. Their publication established a benchmark in the literature by which many assumptions of BFT movements, reproductive behaviour and population structuring has been made. However, during that survey, the researchers, capture protocols and larvae identification methods were the very same that procured the misidentified larvae discussed herein. The possibility that larvae from the Levantine Sea have been misidentified in the past, demands a review of the timing, location and extent to which BFT are spawning in the eastern Mediterranean. If BFT spawning areas are indeed limited in number, then their accurate identification and subsequent conservation from over-exploitation, habitat alteration and pollution is critically important [79].

### DNA extractions and use of molecular markers

It is noteworthy that we were able to extract high quality DNA, and identify to species level, larvae that had been archived in ethanol at room temperature for over 5 years. This possibility should be taken into consideration for all collections containing similarly preserved wildlife specimens. Additionally, the molecular markers used were effective at identifying all larvae to species level. The use of *CO1* for sample identification has been criticized, as it lacks the capacity of discrimination among the Neothunnus tribe and the Pacific and Atlantic BFT [59,62]. Since none of the members of the Neothunnus tribe or *T*. *orientalis* are present in the Mediterranean Sea, this was not a concern for our study. Our reconstruction of NJ trees including the additional non-Mediterranean *Thunnus spp*. sequences were also unable to reliably distinguish the members of that genera, aside from *T*. *alalunga* which consistently clustered independently from the others. Some of the earliest molecular work using a suite of allozymes also found albacore to be most divergent and uncovered very little divergence between *T*. *albacares*, *T*. *maccoyii* and *T*. *orientalis* [80]. DNA sequences from the mitochondrial control region suggest that BFT is a sister taxa of the southern bluefin tuna and that the albacore and Pacific bluefin tuna (PBFT) form a divergent monophyletic clade [60]. The high number of diagnostic loci contained in our *CO1* sequences which discriminate between the albacore and the other Thunnus species support this claim. Clearly, the level of divergence between species is dependent upon the character used for comparison. For example, the high level of divergence between the Pacific and Atlantic bluefin tuna shown when analyzing the mitochondrial control region [60] vanishes when comparing *ITS1* gene sequences [61]. This could be a result of historical hybridization of the PBFT with albacore which resulted in the transfer of the albacore mtDNA genome into the PBFT line [81].

The presence of erroneous or misleading reference sequences in both GenBank and BOLD is both troublesome and concerning. Surely, it was not the intention of the founders of these databases that users should have to check the origin and associated publications of every sequence. The presence of sequences belonging to hybrid organisms in genetic reference databases is confusing and requires additional traceability and documentation.

### BLAST and BOLD assignment

The results generated by BOLD's global alignments and BLAST's local alignments were consistent with one another. The identity similarity threshold used by BOLD prevented the identification of two larvae from the Strait of Sicily, whose identity were later confirmed by BLAST, NJ trees and our character-based identification key. Lowenstein et al. [69] complained that BOLD performed poorly during their attempts to identify species used in sushi but the database was still in its infancy then and it has come a long way since and now contains over 4 million collected from 146 countries. During this period of rapid growth, BOLD began featuring sequences mined from GenBank in their Public Data Portal. We have found several sequences that are in clear violation of the data standards that were established when BOLD was first introduced. The founders of BOLD have clearly stated that sequences do not undergo any kind of centralized review and that the quality of data featured in the database is ultimately dependent upon the individuals that have uploaded the data [48]. At the moment, more than half of the *Thunnus thynnus* sequences featured in BOLD have been mined from GenBank. This hybridization of the two databases, without BOLD's once lauded traceability standards, threatens to undermine the reputation and usefulness of BOLD.

### Neighbour-joining trees

The NJ trees accurately identified larvae to species level for taxa with reference sequences included in the alignments. Clearly, for this approach to work, query larvae must cluster with voucher sequences; an obvious disadvantage when compared to the BLAST and BOLD approaches. The use of NJ trees for species identification has recently come under fire from various sources [49,53]. An entire section focused on NJ trees has been featured in a recent publication entitled “The seven deadly sins of DNA barcoding” [53]. A major disadvantage associated with NJ trees surfaces when individual sequences are assigned to the space between two reference clusters. On these researchers are forced to retreat to lower taxonomic levels [82]. By increasing the number of sequences used for each reference taxa, one can decrease the frequency of these ambiguous outcomes [83]. Our results suggest that the models used for the assembly of distance matrices are also important for the reduction of ambiguous results. Although the K2P model is the most widely used model for NJ tree construction in fish barcoding studies, we have found that distance matrices based on p-distance provide higher BP values. Recent critical reviews of NJ tree construction agree that identification of species using NJ trees based on K2P distances can be inappropriate to the task and more suitable, less complex models can prove more effective [51,52]. In fact, Collins et al. [51], wrote that K2P “was without exception a poorly approximating model at the species level”. Why the K2P model is so widely used in fish barcoding studies is a mystery. Srivathsan and Meier [52] suggest that the widespread use of K2P is a result of its use by early barcoding proponents who wanted to highlight the extreme differences between species. Perhaps researchers are simply following the examples of their peers or that of BOLD which uses the K2P model for their taxa identification trees [48]. Regardless of their weaknesses, phenograms do provide attractive graphic representations of the results.

### Character-based assignment

Realizing the faults inherent in the NJ tree approach, some researchers have found that a character-based method of specimen identification has proven more appropriate to the task [68,84]. Paine et al. [41] constructed a character-based key for identification of degraded tissue samples using reference sequences from 17 species of the Scombridae common to the Western Atlantic Ocean. Our molecular key differs significantly, since the molecular key of Paine et al. [41] begins with position 575 of our alignment, thereby providing only 37 bases for comparison. The molecular key of Lowenstein et al. [69] was generated from alignments shifted only 40 base pair positions towards the 3' end of the *CO1* sequence. The molecular key of Lowenstein et al. [69] includes *Thunnus* species only, as they were making efforts to develop a tool for seafood traceability. Interestingly, they did not include in their study the skipjack tuna, *Katsuwonus pelamis*, a globally cosmopolitan fish in all of the world's oceans and by far the world’s most important tuna fishery. They, too, discovered the same individual nucleotide that distinguishes *T*. *thynnus* from all other tunas. Many of the diagnostic loci discovered by Lowenstein et al. [69] no longer function after the inclusion of the *Scombrus* spp. and *Auxis* spp. sequences. Character-based keys for scombrid species have also been developed for the mitochondrial control region and *ITS1* [60,61].

### Conclusion

Misidentification of early life stage fishes has already occurred in various commercial species, leading to inaccurate estimations of spawning stock biomass [35,85]. We have shown here, for the first time that tuna larvae collected from the Mediterranean Sea have been misidentified by larval survey crews. If the larvae and eggs of BFT are to be used to improve stock assessments in the future, it is imperative that problems associated with species identification are resolved. Fishery independent data, such as larval abundance, are certainly welcome for the betterment of stock assessments where traditional fishery data has lost its credibility; however, scientific rigour and quality control must also accompany this data or we run the risk of repeating the mistakes of the past. Genetic barcoding is a legitimate technique that can support species identification and play a crucial role in fisheries management efforts. We suggest that all routine fisheries work involving larvae should make use of both taxonomists and geneticists in order to ensure both accuracy of results and efficient use of financial resources. We call upon the few taxonomic world experts to update the identification keys associated with fish species of economic and conservation concern, embrace the digital community and pass their knowledge onto new generations through training courses, either *in situ* or with the various Information and Communications Technology platforms now available. Most barcoding efforts are dependent on online databases for voucher sequences; therefore, it is crucial that quality standards are upheld if the barcoding effort is to retain its legitimacy. Enough doubt has been cast on the inappropriate use of NJ trees for specimen identification purposes that they are now regarded by many simply as an attractive communication tool; however, they are still being widely used. Conversely and despite nearly two decades of use in scombrid identification, character-based keys are still not used as a reliable and recognized tool. Perhaps it is the user-friendly automated style of NJ trees that have kept phenograms popular, despite their fallibility. Several efforts are being made to automate the generation of character-based keys and their use as specimen identification tools. The BOLD System now features a Diagnostic Characters analysis suite and R has a package (Spider) dedicated to their use [86]. Character-based keys also have potential for translation into microarrays and high throughput NGS genotyping platforms. Certainly, standardized high throughput genotyping tests will have profound impacts on the future of larvae identification and fisheries surveys.

## Supporting Information

S1 Appendix Reference sequences used for alignments, phenogram analysis and CA development. Unless stated otherwise, all sequences were recovered from BOLD.
(DOC)
