# Supplementary material for: Molecular Identification of Atlantic Bluefin Tuna (Thunnus thynnus, Scombridae) Larvae and Development of a DNA Character-Based Identification Key for Mediterranean Scombrids
Source: PLoS One. 2015 Jul 6;10(7):e0130407. doi: 10.1371/journal.pone.0130407 (PMC4493144; doi:10.1371/journal.pone.0130407)
Supplement: S1 Appendix — Unless stated otherwise, all sequences were recovered from BOLD. (DOC) [file pone.0130407.s001.doc]

S1 Appendix: Reference sequences used for alignments, phenogram analysis and CA development. Unless stated otherwise, all sequences were recovered from BOLD.

Auxis rochei (DSFSF123-09, AB105165.1 [GenBank], AB103468.1 [GenBank], GBGC0053-06, GBGC1666-06, GBGC3356-07, GBGC3358-07, GBGC3359-07, GBGC3360-07, GBGC7423-09)

Auxis thazard (TOBA316-09, FOA810-04, GBGC1669-06, GBGC3350-07, GBGC3351-07, GBGC3354-07, GBGC3355-07, GBGC7414-09, GBGC7415-09, GBGC7416-09)

Scomber colias (FCFPS032-06, AB488406.1 [GenBank], FCFPS117-06, FCFPS118-06, GBGC3320-07, GBGC3321-07, GBGC3322-07, GBGC3323-07, GBGC3324-07, DSFSF201-09)

Scomber japonicus (HQ611117.1 [GenBank], DSFSF202-09, DNATR081-12, MFC235-08, FARG481-08, FARG482-08, FARG486-08, FMV165-08, RFE268-05, RFE269-05)

Scomber scombrus (FCFP095-05, FCFPS040-06, FCFPS041-06, FCFPS152-06, FOA796-04, FOA800-04, SCAFB741-07, SCFAC837-06, SCFAD497-09, GLF063-14

Euthynnus alletteratus ( AB099716.1 [GenBank], BZLWE263-08, CSFOM035-10, DNATR775-13, DNATR775-13, DNATR776-13, DNATR777-13, LIDM537-07, MFSP619-10, MFSP620-10, MXII103-07)

Katsuwonus pelamis (MXII150-07, DSFSF147-09, ANGBF6825-12, ANGBF6832-12, ANGBF6837-12, GBGC1667-06, GBGC4959-08, GBGC4960-08, GBGC4961-08, GBGC4962-08)

Sarda sarda (DNATR075-12, DNATR1220-13, DNATR1221-13, DNATR1223-13, DNATR1239-13, CSFOM065-10, MLFPI110, MLFPI227, MLFPI99, MLFP100)

Thunnus alalunga (FOA868-04, GU256526.1 [GenBank], JN086151.1 [GenBank], CSFOM079-10, GBGCA665-10, GBGCA675-10, GBGCA706-10, RFE231-05, RFE233-05, RFE404-05)

Thunnus albacares (ANGBF6823-12, FOA869-04, FOA870-04, MFC185-08, RFE248-05, RFE250-05, SAFC038-11, SCFAC184-05, TZMSC142-05, WLIND461-07)

Albacore -like Thunnus thynnus (GBGCA443-10)

Thunnus thynnus (FOA947-05, FOA948-05, FOA945-05, DNATR1723-13, DNATR1724-13, GBGC0049-06, GBGC0803-06, GBGCA446-10, SCFAC660-06, SCFAC661-06)

Thunnus maccoyii (FJ605741.1 [GenBank], FOA874-04, FOA875-04, FOA876-04, FOA877-04, GBGCA176-10, GBGCA678-10, GBGCA679-10, GBGCA720-10, GBGCA731-10)

Thunnus obesus (SAFC026-11, ANGBF6817-12, ANGBF6820-12, ANGBF6821-12, FCHIL181-06, FCHIL182-06, FOA881-04, FOA882-04, GBGCA722-10, GBGCA726-10)

Thunnus atlanticus (FOA952-05, MEFM1002-06, MEFM1003-06, MEFM1004-06, MEFM1030-06, MFSP1883-11, MFSP1884-11, MXII115-07, MXII119-07, TOBA079-09)
